# Variational autoencoders can detect coronary artery disease in myocardial perfusion SPECT images

**DOI:** 10.1007/s00259-026-08161-1

**Published:** 2026-08-29

**Authors:** Koichi Okuda, Kenichi Nakajima, Takeshi Hara, Hiroto Yoneyama, Seigo Kinuya

**Affiliations:** 1 Department of Radiation Science, Hirosaki University Graduate School of Health Sciences, 66-1 Hon-cho, Hirosaki-shi, Aomori 036-8564 Japan; 2 Department of Nuclear Medicine, Kanazawa University Graduate School of Medical Sciences, Kanazawa, Japan; 3 Department of Electrical, Electronic and Computer Engineering, Gifu University, Gifu, Japan; 4 Center for One Medicine Innovative Translational Research, Gifu University, Gifu, Japan; 5 Department of Radiological Technology, Kanazawa University Hospital, Kanazawa, Japan

**Keywords:** Variational autoencoder, Myocardial perfusion, Anomaly detection, Imaging

## Abstract

**Purpose:**

Variational autoencoders (VAEs) can detect unsupervised anomalies in medical images. We aimed to determine whether a VAE could automatically detect anomalies in myocardial perfusion SPECT (MPS) images. We then interpreted the training and generative processes of the VAE.

**Methods:**

We trained a VAE on polar maps to learn the variability of normal myocardial perfusion profiles in latent space. The training dataset comprised 3,432 polar map images (49% male) without coronary artery disease (CAD) and the validation set contained 111 polar map images (59% male), among whom, (43% had obstructive CAD). This latent representation allowed the VAE to reconstruct synthetic normal images that served as references to detect anomalies. Abnormal myocardial perfusion was automatically identified by measuring differences between generated and original images using cosine similarity. We also summed stress scores (SSS) using sex-segregated normal databases.

**Results:**

The trained VAE generated reference normal polar maps, regardless of normal and defect profiles and sex differences. The areas under the receiver-operating characteristics curve to detect CAD were 0.89 ± 0.041 for cosine similarity and 0.81 ± 0.030 for SSS (*p* = 0.047). The VAE learned the characteristics of normal male and female myocardial perfusion distributions in the latent space.

**Conclusion:**

This VAE-based approach improved CAD detection in MPS images and eliminated the need to determine sex-specific myocardial perfusion characteristics.

**Supplementary Information:**

The online version contains supplementary material available at https://doi.org/10.1007/s00259-026-08161-1.

## Background

Expert readers have quantified and visually interpreted single-photon emission computed tomography (SPECT) images of myocardial perfusion [1–3]. Summed stress/rest/difference scores and total perfusion deficits are useful for analyzing myocardial perfusion SPECT (MPS) images of patients with coronary artery disease (CAD) [3–8]. Calculated clinical metrics can be compared with a normal myocardial perfusion database (NDB) of patients with a low likelihood of having CAD [3, 7, 8]. Accurate MPS image analysis requires specific NDBs that include sex, population, radiopharmaceuticals, and SPECT imaging conditions [9]. Therefore, we created specific NDBs for sex, race [10–14], stress-rest SPECT acquisition conditions [15], and gamma/cadmium-zinc telluride (CZT) cameras [16].

A convolutional variational autoencoder (VAE) based on deep learning [17, 18], has been applied to detect unsupervised anomalies in medical images. The VAE consists of an encoder, a latent space, and a decoder. The encoder characterizes features of input normal myocardial perfusion images in the latent space during training using a dataset, and the probability of data points is represented as Gaussian distribution. Regardless of whether a patient has a normal or defective perfusion distribution during the generative process, the decoder samples and matches the distribution of patient perfusion in the latent space. Thereafter, it generates a synthetic normal image that exclusively fits the patients. Consequently, abnormal perfusion can be detected by computing differences between input and output images. We initially aimed to detect abnormal myocardial perfusion in MPS images using a VAE. Thereafter, we compared the ability of the VAE model to detect CAD with that of conventional methodology. Moreover, we interpreted the training and generative processes of VAE by comparing conventional quantitative analysis using normal maps.

## Materials and methods

### Normal datasets for training VAE models

We used the Japanese normal databases created by the Japanese Society of Nuclear Medicine working group (JSNM-WG) [10–12] as training datasets of images acquired from 81 males (mean age, height, and weight: 65 ± 11 years, 164 ± 6 cm, and 62 ± 10 kg, respectively) and 87 females (62 ± 13 y, 154 ± 6 cm, and 51 ± 7 kg, respectively). Additional normal images acquired from 70 males and 74 females (mean age, height, and weight: 68 ± 16 y, 165 ± 8 cm, and 66 ± 15 kg; 67 ± 15 y,151 ± 5 cm, and 50 ± 6 kg, respectively) served as training data [15]. The mean age and height of females significantly differed between the datasets (*p* = 0.027 and 0.001, respectively). Stress-rest MPS images were acquired from all patients injected with standard doses between 555 and 1110 MBq ^99m^Tc-sestamibi (MIBI) or ^99m^Tc-tetrofosmin (TF). Details of the JSNM-WG normal database and the characteristics of the patients were published elsewhere [10–12, 15].

### Test dataset for trained VAE model

We retrospectively obtained data from 36 female and 65 male patients with obstructive CAD and a low likelihood of CAD from Kanazawa University Hospital (Table 1). Obstructive CAD was defined as the presence of at least one coronary artery with ≥ 75% stenosis on coronary angiography (CAG). All patients in the obstructive CAD group underwent CAG within 60 days of myocardial perfusion SPECT while in clinically stable condition. The control group consisted of patients with a low clinical likelihood of CAD and did not routinely undergo CAG. However, CAG was performed in eight patients, and coronary stenosis of < 50% and < 25% was observed in three and five patients, respectively. Stress-rest MPS images were acquired from all patients using standard 555–1110 MBq doses of MIBI or TF. The Ethics Committee at Kanazawa University approved this retrospective study (2018 − 269 [3020]), which complied with the ethical guidelines enshrined in the Declaration of Helsinki (2024 amendment). The requirement for informed consent was waived as we retrospectively analyzed innominate data. We applied an opt-out approach and persons who chose not to participate were excluded from analysis.

**Table 1:** Characteristics of patients in test dataset

	Obstructive CAD (*n* = 58)	Low likelihood of CAD (*n* = 43)	*p* values
Female/Male	13/45	23/20	
Age	70.7 ± 9.7	67.6 ± 10.3	n.s.
BSA	1.66 ± 0.21	1.63 ± 0.22	< 0.0001
Angiographic data (≥ 75% stenosis)			
Single vessel	45 (78%)	0	
LAD territory	23	-	
LCx territory	11	-	
RCA territory	11	-	
Double vessel	11(19%)	0	
LAD + LCx	8	-	
LAD + RCA	7	-	
RCA + LCx	7	-	
Triple vessel	2(4%)	0	
Perfusion score			
SSS	8.17 ± 7.48	1.23 ± 1.57	< 0.0001
SRS	4.75 ± 5.91	0.37 ± 0.84	< 0.0001

*LAD* left anterior descending coronary artery, *LCx* left circumflex coronary artery, *RCA* right coronary artery, SSS summed stress score, *SRS* summed rest score

### SPECT image acquisition and data processing

The training dataset consisted of images acquired using GCA9300A (Canon Medical Systems, Otawara, Japan), PRISM 3000XP (Picker Corporation, Cleveland, OH, US), and Symbia T6 (Siemens Healthineers, Erlangen, Germany) gamma camera systems with low-energy high-resolution collimators. The SPECT imaging parameters comprised a 64 × 64 matrix of pixels (5.3 mm for PRISM 3000XP; 6.6 mm for GCA9300A and Symbia T6. The PRISM 3000XP generated 72, and the GCA9300A and Symbia T6 generated 60 projections during circular 360° SPECT acquisition. Energy was centered at 140 keV, with windows of 15% for the Symbia T6 and a 20% for the GCA9300A and PRISM 3000XP. The projection data were then reconstructed using filtered back projection. Attenuation, scatter, and resolution recovery correction were not applied. The cutoff frequency of the Butterworth filter was 0.50 cycles/cm for the GCA9300A and PRISM 3000XP, and 0.51 for the Symbia T6. The order for the Butterworth filter was all eight. The test dataset was acquired using the Symbia T6 to create the training dataset.

Grayscale polar map images acquired under stress and rest were automatically generated by Quantitative Perfusion SPECT (QPS) version 2008.1 (Cedars-Sinai Medical Center, Los Angeles, CA, USA). When the automatic segmentation in the basal myocardium failed during polar map creation, the manual modification of region of interest for the basal myocardium was performed by a single operator. Polar map images were rotated clockwise or counterclockwise by 1° to 5° to augment 1,771 and 1,661 images for females and males, respectively, in the training dataset.

### VAE architecture

**Fig. 1:**
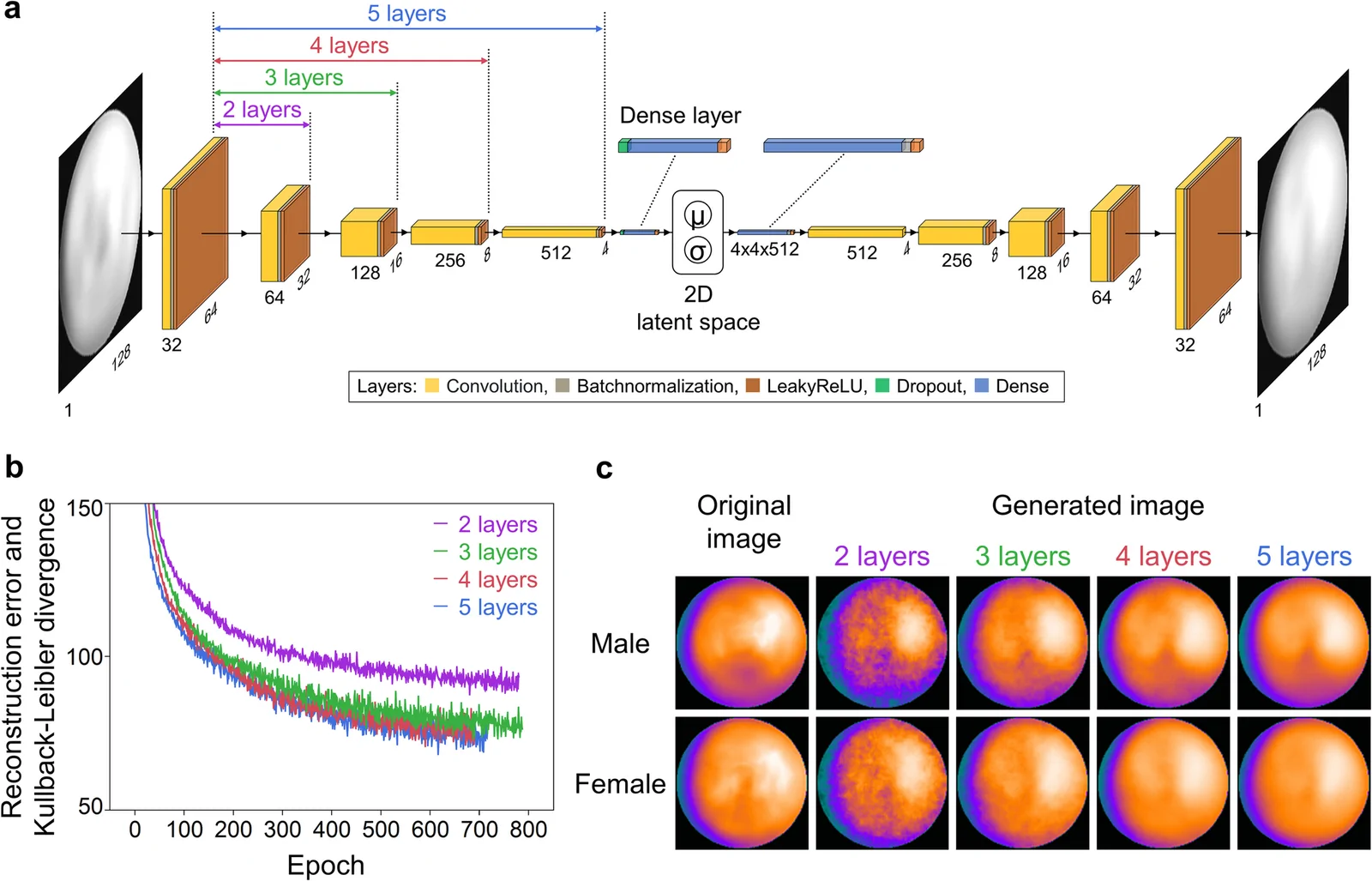
VAE model to generate normal myocardial perfusion images.**(a)** Structure of convolutional VAE with five-encoder and decoder layers. **(b)** Training curves in four VAE models. **(c)** Original and generated images obtained by four VAE models in male and female patients. LeakyReLU, leaky rectified linear unit

We determined the optimal number of layers in standard convolutional VAE architectures with 2–5 layer units (Fig. 1a). The input size of a grayscale polar map image acquired under stress was 128 × 128. Encoding processes consisted of a 2D convolution with a 4 × 4 kernel and stride of 2 followed by batch normalization and leaky rectified linear unit (Leaky ReLU). We determined the first dense layer of neurons received from the previous layer after dropping some neurons (dropout rate) and the number of output neurons (dense output). The loss value during training was computed as the sum of the reconstruction error calculated as mean square error (MSE) and Kullback-Leibler (KL) divergence [19]. Training continued with an early stopping callback that monitored the loss [20], and the VAE stopped after 100 epochs with no improvement in MSE or KL divergence.

### Layer optimization and hyperparameter tuning for VAE model

The optimal number of layer units, which included convolution, normalization, and activation functions, was determined using the convolutional VAE model. The number of dense output, dropout, and learning rates were optimized by the Hyperband hyperparameter tuner [21]. The ranges of hyperparameters were 100–500, 0.0–0.5, and 0.001–0.00001 for the dense output, dropout, and learning rates. Default values were applied to the remaining hyperparameters. The VAE model was implemented using Python 3.7.3, Tensorflow 1.14, and Keras 2.2.4 in Google Colab [22].

### Coronary artery disease detected by VAE

Abnormal perfusion can be detected by computing differences between the input and output polar map images. Differences were evaluated using MSE, mutual information (MI), structural similarity index measures (SSIM), peak signal-to-noise ratios (PSNR), dice coefficients, and cosine similarity. We compared the best performance metric analyzed using the area under the receiver operating characteristics (ROC) curves (AUC) with summed stress scores (SSS) to detect CAD. All SSSs were automatically calculated using QPS software based on the Japanese normal databases created by the Japanese Society of Nuclear Medicine working group [9–12].

### Interpretation of the VAE training and generative processes

Using the training datasets, the distributions of male and female data points were visualized in the 2D latent space to interpret the VAE training process. Polar map images were generated by decoding individual points in the latent space, and the generated myocardial perfusion distribution patterns were visually evaluated. In addition, the mean and deviation normal databases were created using rest polar maps from 43 patients with a low likelihood of CAD (summed rest score < 4) in the test dataset. Moreover, another set of mean and deviation normal databases was created using synthetic polar maps representing normal myocardial perfusion generated by the VAE from the stress polar maps in the test dataset. These normal databases derived from original polar maps and VAE-generated synthetic polar maps were visually compared.

### Statistical analysis

Continuous and non-parametric variables are expressed as means ± standard deviation (SD), and medians, respectively. Differences in continuous variables were analyzed using the Student t-tests and Wilcoxon signed-rank tests. All statistical tests were two-tailed, and values with *p* < 0.05 were considered significant. We calculated MSE, MI, SSIM, PSNR, dice coefficient, and cosine similarity using the scikit-learn machine learning library [23]. All data were statistically analyzed using JMP version 11.2.1 (SAS Institute Inc., Cary, NC, USA).

## Results

To develop the optimal VAE models, we searched for the hyperparameters that yielded the lowest loss values (Supplementary Fig. 1). The optimized hyperparameter values of dense output, dropout rate, and learning rate were, respectively, 150, 0.2 and 0.0001 for two-layer; 260, 0.1 and 0.0001 for three-layer; 210, 0.3 and 0.0001 for four-layer; 270, 0.3 and 0.0001 for five-layer models. Compared with a loss of 93.1 at 690 epochs in the two-layer model, 74.8, 75.1, and 76.5 were derived from the three-, four-, and five-layer models, respectively (Fig. 1b). Image quality was degraded in the two-layer model (Fig. 1c), which was excluded from further evaluation.

**Fig. 2:**
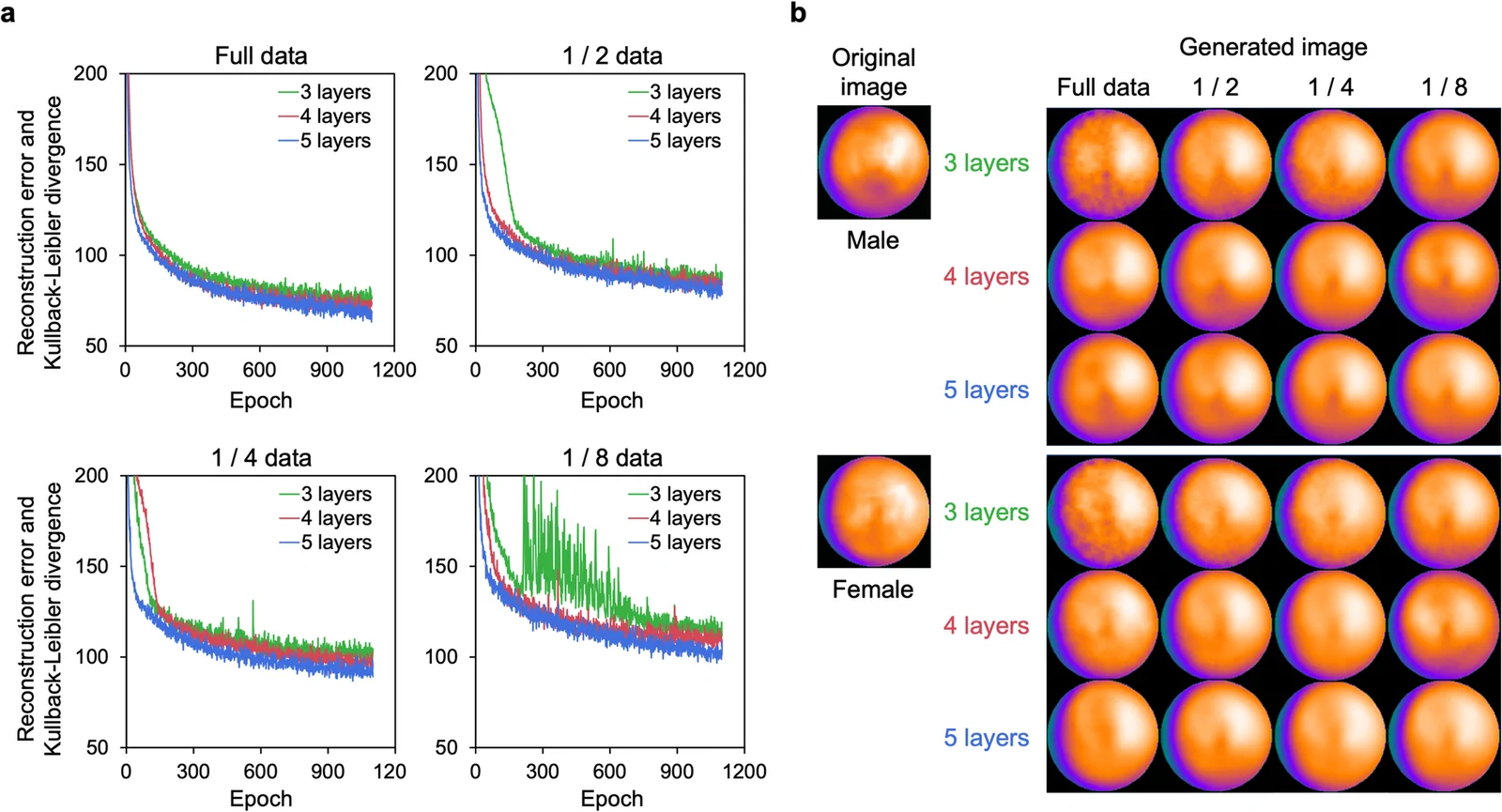
Effects of sample size on training VAE models.**(a)** Curves of training datasets in four VAE models. **(b)** Original and generated images of male (upper) and female (lower) patients

To evaluate the influence of training dataset size on VAE performance, we compared the loss values of the three-, four-, and five-layer models using training datasets of full, half, quarter, and one-eighth size (Fig. 2). The optimized hyperparameters with the lowest loss values were used in the four training datasets (Supplementary Figs. 2–4). At 1100 epochs, where losses were higher in the one eighth dataset, whereas values were lower in the full, half, and quarter datasets (Fig. 2a). The loss values for the five-, four-, and three-layer models were 69.0, 74.0, and 76.9 in the entire dataset, respectively; 80.2, 83.5, and 90.4 in the half dataset; 89.1, 95.5, and 104.6 in the quarter dataset; and 108.5, 109.1, and 111.7 for the one-eighth dataset. Nevertheless, the quality of the generated images was stable among the datasets (Fig. 2b).

**Fig. 3:**
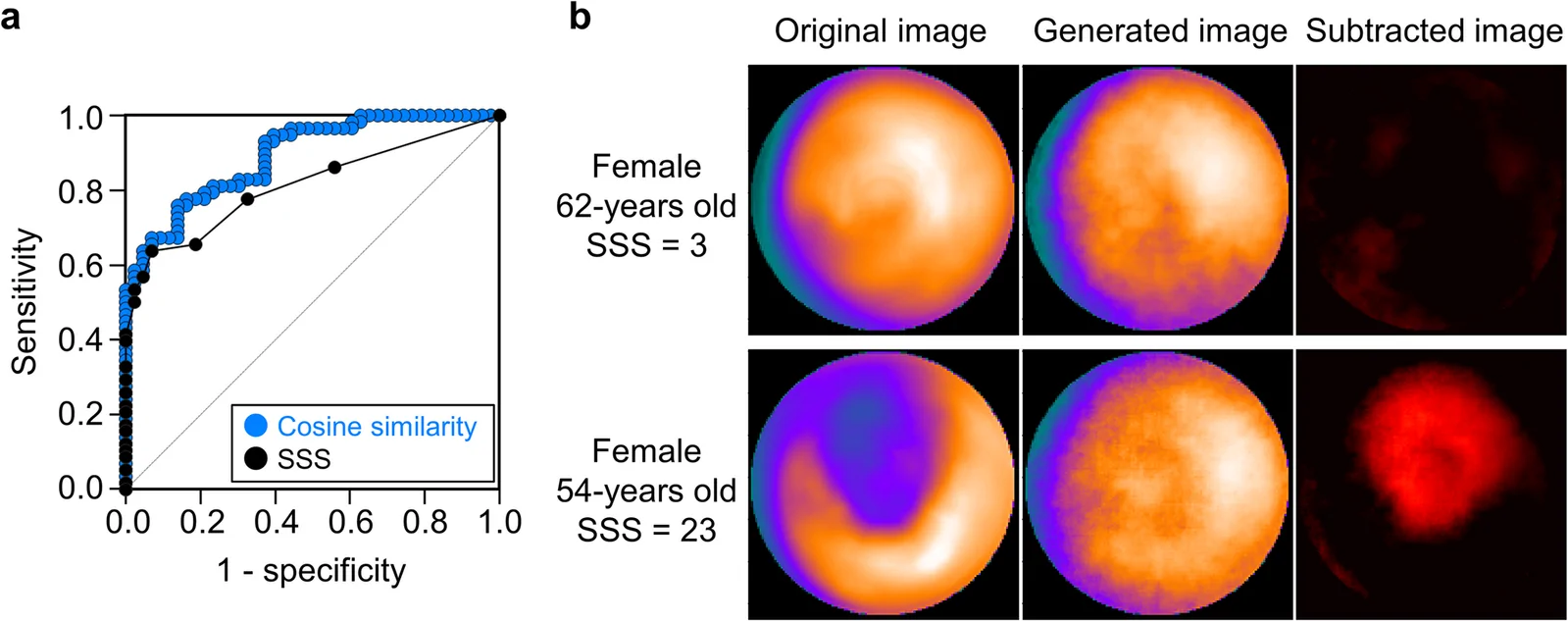
Detection of CAD using trained VAE models **(a)** AUC values for MSE, SSIM, dice coefficient, MI, PSNR, and cosine similarity in three-, four-, and five-layer models. **(b)** ROC curves comparison of cosine similarity and SSS. **(c)** Original and generated images of normal female (upper) and patient with ischemia at LAD region (lower). Generated images were created from original images using the 3-layer VAE model (Epoch; 1000). The pixel values of subtracted images were calculated as originals minus generated images

When the VAE models with three, four, and five layers were trained using full datasets, the AUC of MSE, SSIM, dice coefficient, MI, PSNR, and cosine similarity remained stable during 100 and 1100 epochs (Supplementary Figure. 5). The averaged AUC values of six metrics were 0.850, 0.828, and 0.824 for the three-, four-, and five-layer models, respectively. The highest AUC of cosine similarity derived from the three-layer model at 1000 epochs was significantly higher than that of SSS (0.892 ± 0.0412 vs. 0.814 ± 0.0301, *p* = 0.047; Fig. 3a). Figure 3b shows two patients with normal perfusion and anterior ischemia. The generated perfusion distribution was similar to that of the original in the patient with normal perfusion. Moreover, the VAE model generated a synthetic normal perfusion distribution from the hypoperfusion image. Subtracted images clearly delineated an ischemic area in the apex to anterior wall.

**Fig. 4:**
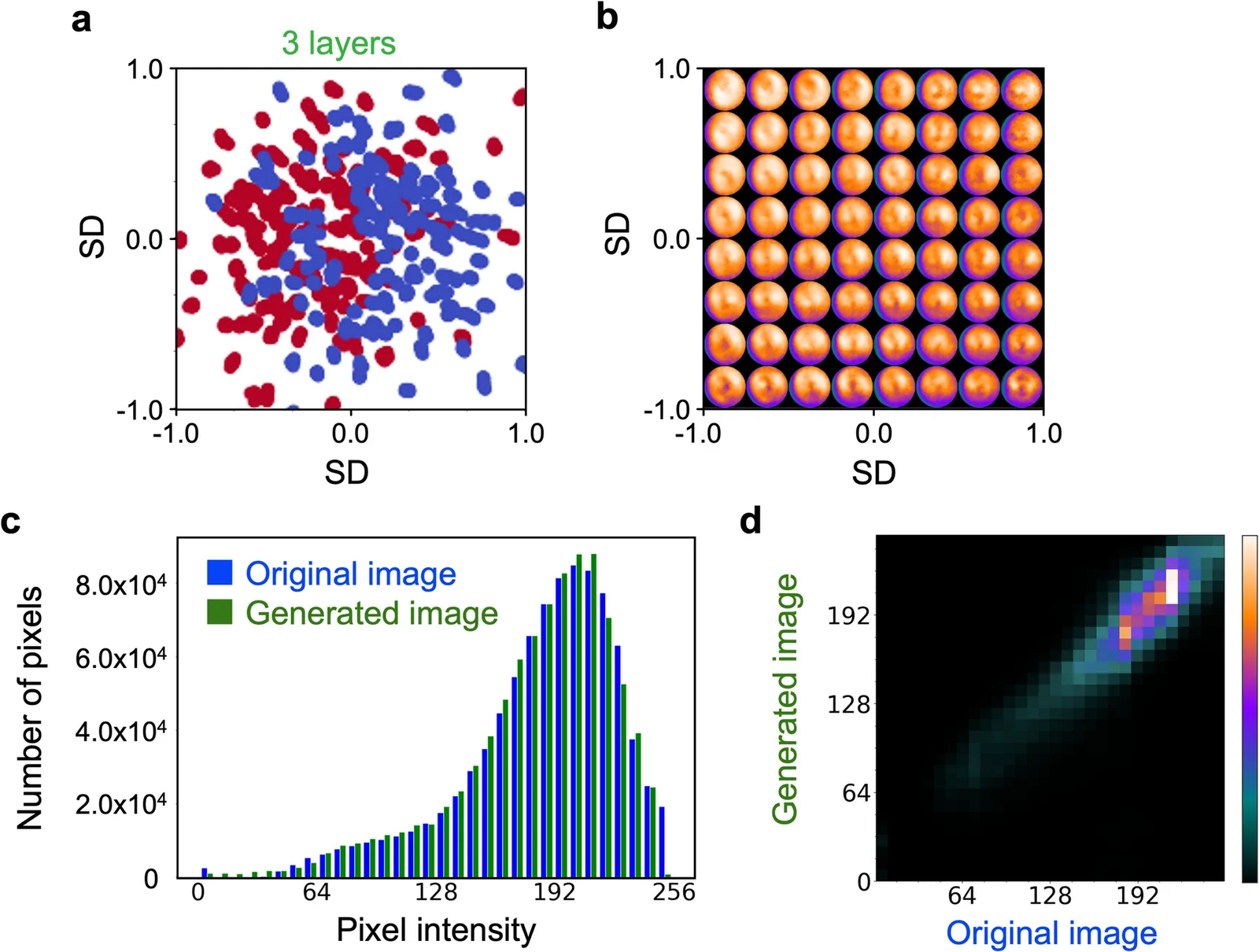
Interpreted VAE model in MPS image analysis **(a)** Visualization of latent space distribution in males (blue) and females (red). **(b)** Polar map representation derived from the latent space. **(c)** Histograms of original (blue) and generated (green) pixels in patients with normal distribution. **(d)** Joint distribution of original and generated pixel values visualized as a 2D histogram (*r* = 0.87, *p* < 0.0001). SD, standard deviation

To interpret the training and generative processes of the VAE, conventional quantitative analysis using normal polar maps and latent space analysis were examined. Distributions of normal myocardial perfusion in males and females were mapped in the 2D latent space (Supplementary Fig. 6). The ranges of data variation in the X and Y axis directions (X, Y) were (−1.2-1.5, −1.1-1.5) for the 3-layer, (−1.9-1.7, −2.0-2.1) for the 4-layer, and (−2.5-2.4, −2.6-2.6) for the 5-layer models. The distances between centroids of male and female data points were stable at 0.57, 0.67, and 0.46 in the 3-layer, 4-layer, and 5-layer models. Although some male and female data points were overlapped, they were respectively distributed from the center to the right and left (Fig. 4a). These findings suggested that the VAE latently represented sex-related differences in myocardial perfusion distributions in the latent space. The polar maps showed that myocardial perfusion was uniform in the upper left corner of the latent space, while that at the inferior was decreased in the lower right corner (Fig. 4b). These respective profiles corresponded to normal myocardial perfusion distributions in males and females. We compared pixel values between original and generated images, and found similar histograms (dice coefficient = 0.61, Fig. 4c). The joint distribution of pixel values between the original and generated images showed good agreement (Fig. 4d). When the mean and deviation normal databases were generated using the test dataset, mean maps were visually equivalent between original and VAE-generated normal databases (Supplementary Fig. 7). The deviation maps derived from original and generated data were partly agreed.

## Discussion

The main findings of this study were that the VAE model facilitated the detection of abnormal myocardial perfusion distribution in polar map images. The VAE model outperformed the conventional method of detecting CAD. The latent space and generated polar images were helpful to interpret the training and generative processes of VAE.

Deep learning has recently been applied to evaluate MPS images. It can predict obstructive diseases and classify cardiac sarcoidosis as well as myocardial perfusion distribution [24–26]. However, deep learning was used not only for prediction but also for classification, rather than for image generation in these studies. The novelty of our study lies in the application of deep learning–based image generation to create a normal myocardial distribution. Moreover, quantitative evaluation was achieved by comparing myocardial perfusion in patients with a synthetic normal myocardial perfusion distribution using the VAE.

Coronary artery disease was detected in VAE output images with low, rather than high resolution. We previously calculated myocardial perfusion using segmental SSS in a 17-segment model [6]. Here, we calculated segmental SSS based on all, and averaged pixel values in segments and four sections of sub-segments. The sub segmental calculation delivered the best results. Visual interpretation of MPS images might identify changes in whole myocardial perfusion images, but overlook small changes.

We considered whether the VAE could automatically determine myocardial perfusion distribution in images acquired from males and females. Generally, inferior and anterior myocardial perfusion counts are respectively decreased in males and females [11]. Figure 4 indicated that the VAE specifically clarified a sex difference in inferior myocardial perfusion distribution in the latent space. Conventional quantitative analyses were based on sex-segregated normal databases. However, the characteristics of male and female myocardial perfusion did not need to be considered using our VAE-based approach.

Differences in gamma camera systems, acquisition protocols, and reconstruction methods may alter image characteristics and consequently influence the VAE performance. In particular, differences in detector number and configuration, system design, spatial resolution, and sensitivity among camera systems may affect myocardial perfusion distribution [27]. Although CZT cameras were not used in the present study, image characteristics might also differ between conventional Anger and CZT cameras [16]. Acquisition protocols may also affect image characteristics, as 180-degree and 360-degree SPECT acquisitions can produce different myocardial perfusion patterns [10–12]. The present study adopted 360° acquisition because it provides a more uniform representation of the entire myocardium with reduced angular sampling bias, which is advantageous for quantitative image analysis. In contrast, 180° acquisition provides higher count sensitivity for the anterior wall and shorter acquisition times. Since these acquisition-specific myocardial perfusion patterns are reflected in normal databases, a VAE trained on normal myocardial perfusion images is expected to learn protocol-dependent characteristics. Therefore, further studies using 180° acquisition data will be important to determine whether separate training or protocol-specific models are required. To minimize variability in image characteristics, the present study employed standardized imaging conditions, including conventional Anger cameras, 360° acquisition, and filtered back-projection reconstruction.

The applicability of our VAE approach to other radiotracers, such as ^123^I-labeled tracers, remains to be investigated. Differences in photon energy, scatter characteristics, and collimator performance may affect image quality and myocardial perfusion distribution patterns. In particular, collimator selection is a key factor for ^123^I imaging because high-energy photon penetration will influence image quality. If large and diverse training datasets of myocardial perfusion images acquired with ^123^I-labeled tracers using different collimators were available, the VAE might potentially learn tracer- and collimator-specific myocardial perfusion distribution patterns and generate normal reference images.

Our study has several limitations. First, we only used polar map images rather than original SPECT images. Clinical interpretation of myocardial perfusion imaging is based not only on polar maps but also on the original SPECT images. Furthermore, converting the three-dimensional myocardial perfusion distribution into a two-dimensional polar map may result in the loss of spatial information from the myocardial walls. Although quantitative assessment using SPECT polar maps is a standard approach for evaluating myocardial perfusion defects, a VAE trained directly on the original SPECT images may provide different diagnostic performance. Therefore, a VAE-based framework that integrates both polar maps and original SPECT images may provide a more comprehensive analysis. Second, we did not optimize the sample size for training data, and the clinical validity of generative polar map images was not evaluated. Synthetic normal perfusion distribution generated by the VAE should be further explored. Third, since all polar map images were reconstructed using filtered back projection, their application to the ordered subset expectation maximization remains unclear.

## Conclusions

The convolutional VAE can improve CAD detection in MPS images. The VAE automatically learned the characteristics of myocardial perfusion distribution in normal males and females in the latent space, and estimated sex-specific normal perfusion distributions in generative polar map images.

## Supplementary Information

Below is the link to the electronic supplementary material.

Supplementary Material 1 (DOCX 9.71 MB)

## Data Availability

The image datasets are not publicly available due to the approval of Ethics Committee but are available from the corresponding author on reasonable request.
