# Supplementary material for: Variational autoencoders can detect coronary artery disease in myocardial perfusion SPECT images
Source: Eur J Nucl Med Mol Imaging. 2026 Aug 29;53(13):8014–22. doi: 10.1007/s00259-026-08161-1 (PMC13727861; doi:10.1007/s00259-026-08161-1)
Supplement: Supplementary file 1 [file 259_2026_8161_MOESM1_ESM.docx]

**Supplementary data**

**Variational autoencoders can detect coronary artery disease in myocardial perfusion SPECT images**

Koichi Okuda	Kenichi Nakajima	Takeshi Hara

Hiroto Yoneyama	Seigo Kinuya

**Supplementary Figure 1.** Combinations of optimized hyperparameters in the VAE model with two (a), three (b), four (c), five (d) layers.

**Supplementary Figure 2.** Combinations of optimized hyperparameters for the VAE models with three (a), four (b), and five (c) layers in half training dataset.

**Supplementary Figure 3.** Combinations of optimized hyperparameters for the VAE model with three (a), four (b), and five (c) layers in quarter training dataset.

**Supplementary Figure 4.** Combinations of optimized hyperparameters for the VAE model with three (a), four (b), and five (c) layers in one eighth training dataset.

**Supplementary Figure 5.** CAD detection performance of VAE model using six metrics

AUC values for MSE, SSIM, dice coefficient, MI, PSNR, and cosine similarity in three-, four-, and five-layer VAE models. Six evaluation metrics were categorized into three groups: pixel-wise similarity (MSE and PSNR), structural similarity (SSIM and dice coefficient), and distributional or feature similarity (MI and cosine similarity). MSE, mean square error; MI, mutual information; SSIM, structural similarity index measures; and PSNR, peak signal-to-noise ratios.

**Supplementary Figure 6.** Visualization of the latent space distribution in male and female groups.

**a**. Individual data points of males (blue) and females (red) in the three-layer (upper), four-layer (middle), and five-layer (lower) models. **b**. Polar maps generated by decoding latent variables sampled from the latent spaces. Since the latent space was modeled as a Gaussian distribution, the x- and y-axis values indicated the number of standard deviations (SDs) from the distribution center. SD, standard deviation.

**Supplementary Figure 7.** Interpretation of VAE model in normal databases.

Mean (left) and deviation (right) normal databases generated from the 43 patients with a low likelihood of CAD (summed rest score < 4) in the test dataset. The original normal databases were created from the rest polar maps, whereas another set of normal databases was created using synthetic polar maps generated by the VAE from the stress polar maps.
